# Prevalence, Surgical Anatomy, and Clinical Significance of the Thyroidea Ima Artery in Thyroidectomy: A Systematic Review

**DOI:** 10.7759/cureus.110102

**Published:** 2026-06-02

**Authors:** Dimitrios Bartziotas, Iraklis Mpartziotas, Dimosthenis Chrysikos, Ameer Shehade, Theodore Troupis

**Affiliations:** 1 Department of Anatomy, School of Medicine, National and Kapodistrian University of Athens, Athens, GRC; 2 Department of Plastic Surgery, General Oncological Hospital of Kifisia "Oi Agioi Anargyroi", Athens, GRC

**Keywords:** anatomical variations, arteria thyroidea ima, neck surgery, thyroidea ima, thyroidectomy, thyroid ima artery

## Abstract

The thyroidea ima artery (TIA) is an unpaired anatomical variant of the thyroid arterial supply that ascends along the anterior surface of the trachea to the inferior pole of the thyroid gland or the isthmus. Despite long-standing anatomical interest in this vessel, a comprehensive and up-to-date synthesis of the evidence on its prevalence, surgical anatomy, and clinical implications in thyroidectomy is still lacking. This systematic review was conducted in accordance with the Preferred Reporting Items for Systematic reviews and Meta-Analyses (PRISMA) 2020 guidelines. A comprehensive search of PubMed/MEDLINE (Medical Literature Analysis and Retrieval System Online) was performed, covering the period from January 2010 to December 2025. Out of 54 identified records, 20 met the inclusion criteria and were included in the analysis, comprising 14 case reports, four observational studies based on computed tomography angiography (CTA), and two cadaveric or mixed cadaveric-imaging series, originating from 10 countries. Across the included studies, the reported prevalence of TIA ranged from 0.1% to 5.71%, with cadaveric studies consistently showing a higher detection rate than imaging-based studies. The brachiocephalic trunk was the most frequently reported arterial origin overall, although the aortic arch predominated in certain Caucasian populations, indicating notable population variability. Co-existence of the TIA with unilateral or bilateral inferior thyroid artery deficiency was a recurrent observation, with direct implications for the risk of inadvertent parathyroid devascularization and postoperative hypoparathyroidism. The relationship between the TIA and the recurrent laryngeal nerve remains poorly characterized, although close parallel courses have been described and warrant particular surgical attention. The risk of hemorrhagic complications from unrecognized TIA injury during thyroid, tracheal, or mediastinal surgery is significant, given the vessel's origin from high-pressure great arteries; transcatheter arterial embolization may serve as a safe adjunct for hemorrhage prevention and selective thyroid devascularization in specific clinical scenarios. This review highlights the considerable anatomical variability of the TIA and supports a systematic preoperative vascular assessment, including CTA with three-dimensional reconstruction when feasible, particularly in patients with substernal goiter, known inferior thyroid artery deficiency, or aortic arch anomalies, prior to thyroid or anterior neck surgery.

## Introduction and background

The thyroidea ima artery (TIA) owes its name to Latin i, which means lowest, and this is where it fits in the neck anatomy of surgery. This vessel was first described by Johann Ernst Neubauer in 1772 and then by Pratt in 1916, who described it in more detail [1]; it has, over two centuries since then, attracted the interest of anatomists and surgeons alike. The TIA is also referred to as the thyroid artery of Neubauer, an anatomical variant of the cervical arterial supply, which, although rare, has a significant clinical impact when it is seen at the wrong time, during surgery. Even the fact that it has been repeatedly discovered again and again during cadaveric dissections, radiological series, as well as intraoperative reports over a hundred years long, is testimony to the continued difficulty that it places on those who work in the anterior neck.

The thyroid gland lies in the visceral compartment of the anterior neck, between the levels of the C5 and T1 vertebrae, and consists of two lateral lobes joined by an isthmus that crosses the second to fourth tracheal rings. In normal conditions, its arterial supply comes from two paired vessels, the superior thyroid artery, which is the first anterior branch of the external carotid artery and feeds the upper pole, and the inferior thyroid artery, which arises from the thyrocervical trunk and supplies the lower pole, most of the posterior surface of the gland, and the parathyroid glands. Beyond this, a few non-pathological anatomical variations are encountered often enough that they should be familiar to anyone operating in this region. The most frequent of these is the pyramidal lobe, an upward midline projection from the isthmus that represents the persistent caudal end of the thyroglossal duct. A recent meta-analysis of 24 studies estimated its pooled prevalence at 42.82% [2]. Variations of the inferior thyroid artery itself, both in origin and course, as well as its unilateral or bilateral absence, are also seen. It is exactly within this background of variable arterial anatomy that the thyroidea ima artery becomes clinically relevant, both as an additional source of supply to the lower pole and as a finding that frequently appears together with the absence of the inferior thyroid artery.

Embryologically, TIA is not a pure anomaly but rather a remnant of the extensive vascular network that serves the developing thyroid gland in fetal development. In the third to seventh gestational week, a large plexus of arteries is involved in the descent and differentiation of the thyroid gland, most of which then contract, and the final superior and inferior thyroid arteries develop. The TIA has been thought to reflect the continuation of one of these embryological routes, namely that of the major blood supply to the third and fourth pharyngeal pouches and the ultimobranchial body. One suggestion made by Robinson that can be especially useful is that hemodynamic changes that take place in the environment of conotruncal cardiac abnormalities can prefer the untimely involution of the TIA, connecting its destiny with the overall cardiovascular development [3]. The mechanisms behind the maintenance of this vessel into adult life in some people and its involution in the great majority are not fully understood, but anthropological and ethnic influences seem to be at play here, with Asian individuals showing a higher rate than Caucasian populations [4].

There is also a great range in the prevalence of the TIA reported in the literature, with prevalence reported as less than 0.1 to more than 17% based on the population under study and the methodology used [5]. A major systematic review and meta-analysis identified a total prevalence rate of about 3.3 in adults, with cadaveric dissection studies having a higher prevalence rate compared to imaging-based studies, which is probably due to a difference in the methodology of detection and not necessarily to biological variation [5]. It is a single vessel that runs anteriorly along the trachea to the inferior pole of the thyroid gland or the isthmus and has a diameter of 2-5 mm in diameter [6]. It sometimes produces two or three branches, and instances of a twofold TIA have been recorded, only making the anatomical picture an already unstable one even more complex [6,7]. Its source is also not predictable, with the brachiocephalic trunk being the most frequent, but also the subclavian artery, aortic arch, left or right common carotid artery, subclavian artery, internal thoracic artery, thyrocervical trunk, and other vessels [7,8]. The prevalence of the TIA in a large percentage of cases is associated with the unilateral or bilateral absence of the inferior thyroid arteries, which might indicate a type of compensation where the TIA has taken the place of the missing vessel [9,10].

TIA is not necessarily localized to the thyroid gland only. Branches have been seen to serve the infrahyoid muscles, the sternoclavicular joints, the thymus, the parathyroid glands, and even the enlarged parathyroid adenoma, a discovery with significant consequences in relation to parathyroid arteriography and localization studies [11,12]. Such discrepancy in supply territory implies that the artery can be found at unpredictable sites during any surgical exploration of the anterior neck, mediastinum, or superior thoracic aperture, not simply during thyroidectomy, but also during tracheostomy, cricothyrotomy, and mediastinal surgery [12,13]. Inadvertent TIA has a hemorrhagic risk because the vessel is normally high-pressure arterial in nature, as the brachiocephalic trunk or aortic arch, and thus laceration during surgery can result in rapid and possibly uncontrollable bleeding, which may require urgent sternotomy [14].

Although the literature of case reports and anatomical studies is increasing, there is still no systematic synthesis of the evidence on the occurrence, surgical anatomy, and clinical relevance of the TIA in the thyroidectomy-specific scenario. The current systematic review aims to cover this gap by critically reviewing articles that have been published in the 2010-2025 period and will offer an organized report on the existing body of knowledge and provide clinically relevant advice to surgeons who work in thyroid and neck surgery.

## Review

Materials and methods

This systematic review was conducted according to the Preferred Reporting Items for Systematic Reviews and Meta-Analyses (PRISMA) 2020 guidelines [15].

Search Strategy

In January 2026, a thorough search was conducted on the PubMed/MEDLINE (Medical Literature Analysis and Retrieval System Online) electronic database that encompassed the years from January 2010 to December 2025. No extra filters for language, publication type, or subject were used. The search query was as follows: thyroidea ima[All Fields] OR "thyroid ima artery"[All Fields] OR "arteria thyroidea ima"[All Fields]. Language restrictions were applied (only articles in English were considered eligible).

A total of 54 records were retrieved in this search. All identified records were screened by two investigators (DB and IB) using the title and abstract. Titles of interest were further reviewed by abstract. Finally, reference lists of eligible studies were manually assessed in order to detect any potential relevant articles (“snowball” procedure). No Greek-language or other non-English sources were consulted at any stage of this review, and no translation tool was used at any point of the search, screening, data extraction, or synthesis process. All potentially eligible studies were retrieved by accessing their full-text articles, and final decisions of inclusion were made individually by the two reviewers. The dispute was solved by means of discussion and agreement with a third reviewer. 

Eligibility Criteria

The inclusion criteria were studies based on human subjects, adult and fetal cadaveric material, cross-sectional observational studies, prospective or retrospective studies, case reports, or case series. Study design must include cadaveric dissection studies and must report one or more of the following outcomes: the prevalence of the TIA, its arterial origin or anatomical path, or its clinical and surgical relevance in the neck or thyroid surgery. Only articles that were published in English were included.

The exclusion criteria were as follows: systematic reviews, narrative reviews, and meta-analyses, which were utilized to provide background information only; animal studies; studies where the TIA was only mentioned incidentally and no anatomical or clinical data was provided; genetic or chromosomal studies where the TIA was discussed as a secondary finding and by-product; and articles that did not state the names of the authors. Articles that had been published prior to January 2010 were not included in the formal synthesis, but were included in the Introduction and Discussion where appropriate, due to the foundational significance of some of the older anatomical reports.

Data Extraction

Two reviewers (DB and IB) extracted data based on a standardized data collection form. The variables that were tabulated per study included the first author and the year of publication, country of origin, study design and methodology, sample size and subject characteristics, reported TIA prevalence, arterial origin of the TIA, anatomy course and branch pattern, association with other structures such as recurrent laryngeal nerve and inferior thyroid arteries, other anatomical variants, and clinical/surgical meaning as described by the authors. A third reviewer then cross-checked the extracted data as a way of confirming accuracy and complete data.

Quality Assessment

The methodological quality of the included studies was independently assessed by two reviewers (DB and IB) using the Joanna Briggs Institute (JBI) Critical Appraisal Tools, applied according to study design. Case reports were assessed with the JBI Critical Appraisal Checklist for Case Reports (eight items) [16], case series with the JBI Critical Appraisal Checklist for Case Series (10 items) [17], and CTA-based observational studies with the JBI Critical Appraisal Checklist for Analytical Cross-Sectional Studies (eight items) [18]. Each item was rated as yes, no, unclear, or not applicable. A study was classified as low risk of bias when at least 75% of the applicable items were rated as yes (≥ 6/8 for case reports and cross-sectional studies, ≥ 7/10 for case series), as moderate risk when 50-74% of items were met, and as high risk when fewer than 50% were met.

Disagreements between the two reviewers were resolved through discussion and, when needed, with the involvement of a third reviewer. One included item, an editorial eComment by Barbetakis et al. [14], does not contain individual patient demographics, clinical timeline, diagnostic methods, or follow-up, and for this reason, a formal JBI appraisal could not be applied to that, and it was retained in the synthesis as supportive surgical commentary only. No quantitative synthesis, meta-analysis, or statistical pooling was performed given the heterogeneity of study designs and operational definitions of the thyroidea ima artery across the included studies.

Results

Article Selection and Study Demographics

**Figure 1:**
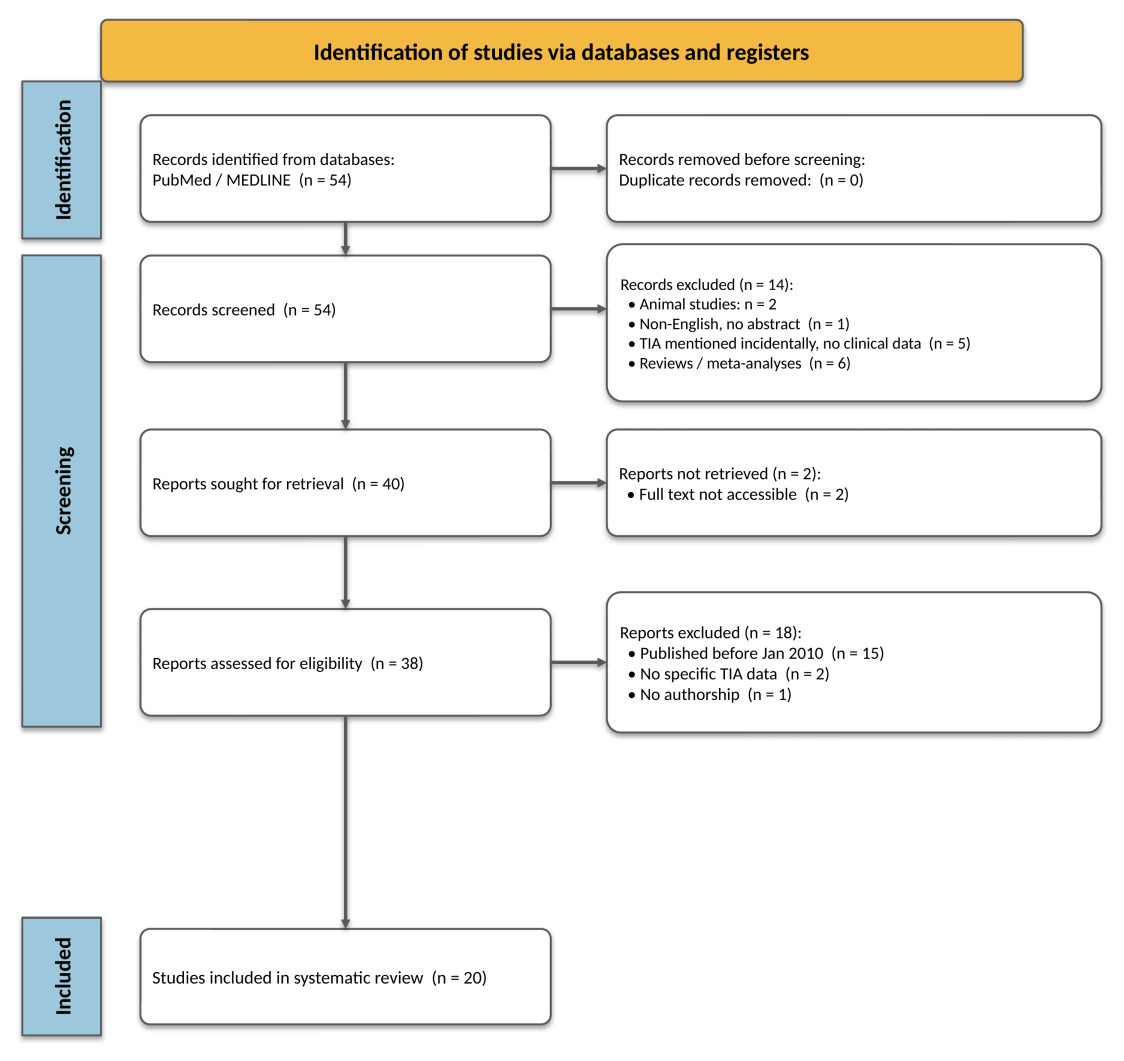
PRISMA 2020 flow diagram of study selection TIA: thyroidea ima artery; PRISMA: Preferred Reporting Items for Systematic reviews and Meta-Analyses

The search strategy retrieved 54 articles that were evaluated for full-text evaluation. The search strategy is depicted in Figure 1. At the screening stage, 14 were eliminated due to the following reasons: two animal studies, one non-English record without an available abstract, five studies where TIA was only noted incidentally without anatomical or clinical information, and six were reviews or meta-analyses. The rest of the 40 records were recalled in order to be assessed in full-text. Two of these were not fully retrieved in text and were therefore excluded. After the full-text inspection of the other 38 records, 18 were filtered out (15 were published prior to January 2010, two had made the TIA a secondary finding only, and one had no identifiable author). The PRISMA 2020 flow diagram (Figure 1) summarizes the choice of the study. Twenty studies [7,8,13,14,19-34] were deemed eligible and were included in the analytic cohort. Overall, the studies encompass a total of 2,295 patients who have been included in this systematic review.

The 20 selected publications were published between 2014 and 2025 and had their origin in 10 countries in Europe, Asia, and North America (Greece, Turkey, Slovakia, India, Japan, Bulgaria, the United States, Romania, Nepal, and China). Most of the literature included was in the form of a case report (n = 14), followed by CT- or CTA-based retrospective or prospective observational studies (n = 4) and cadaveric or mixed cadaveric-CTA studies (n = 2). Table 1 provides a summary in detail of the characteristics and main findings of each of the studies included.

**Table 1:** Characteristics of included studies (n = 20) CCA: common carotid artery; ITA: inferior thyroid artery; RLN: recurrent laryngeal nerve; TIA: thyroidea ima artery; AA: aortic arch; ARSA: aberrant right subclavian artery; TAE: transcatheter arterial embolization; SETA: selective embolization of thyroid arteries; CTA: computed tomography angiography; BCT: brachiocephalic trunk; SCA: subclavian artery; RCCA: right common carotid artery; STA: superior thyroid artery; MTA: middle thyroid artery

Study (Author, Year)	Country	Study Type	Sample	TIA Incidence / Prevalence	TIA Origin	Course & Branches	Surgical Significance	RLN Relationship
Barbetakis et al., 2014 [14]	Greece	Case report (editorial eComment)	1 patient	Cited: 3–10%	Background literature cited: brachiocephalic trunk (most common); aortic arch, right CCA, subclavian, internal thoracic (less frequent) — specific origin not reported in this case	Ascends anterior to trachea to inferior thyroid pole	Substernal goiter thyroidectomy — digital mediastinal dissection may lacerate an unidentified TIA, causing major haemorrhage. Urgent sternotomy may be required if source is not controlled.	Not mentioned
Karacan et al., 2014 [19]	Turkey	CT angiography retrospective study	1,000 patients	0.1% (1/1,000)	Aortic arch (only origin reported in this series)	Not detailed	Recognition important for aortic/endovascular procedures and neck surgery	Not mentioned
Natsis et al., 2016 [32]	Greece	Cadaveric study + literature review (267 cadavers)	267 cadavers; TIA case: female, 37 yr	0.4% (1/267) — TIA coexisting with ARSA	Right CCA (lower middle third); coexisted with aberrant right subclavian artery (ARSA)	Ascending from lower middle third of RCCA; coursed upwards to supply right thyroid lobe and lower isthmus	Coexistence of ARSA + TIA + non-recurrent RLN — combination of high clinical relevance for thyroidectomy, parathyroidectomy, and endarterectomy	Right non-recurrent laryngeal nerve coexisted (ARSA-related); not directly related to TIA course — no spatial measurement reported
Kamparoudi et al., 2016 [13]	Greece	Clinical case report	1 patient, male, 66 yr	Cited: 3–10%	Not specified in this case	Anterior to trachea	TIA injury during percutaneous dilatational tracheostomy → severe haemorrhage → converted to open surgical tracheostomy; bleeding controlled by ligation; patient survived. Authors note urgent sternotomy would have been required had ligation failed.	Not mentioned
Lovasova et al., 2017 [24]	Slovakia	Case report (cadaveric)	1 cadaver, female, 72 yr	Not reported (single case)	Brachiocephalic trunk (BCT)	Branched from ventromedial surface of BCT; coursed upright to inferior thyroid isthmus. Length: 17.8 mm; diameter: 3.3 mm.	Coexistence of 5 thyroid arteries on one side (STA, ITA, TIA, middle thyroid artery, aberrant accessory ITA) — importance for radical neck dissection and minimizing postoperative complications	RLN followed normal course behind ITA; no close relationship to TIA or variant vessels documented
Esen et al., 2018 [8]	Turkey	CT angiography retrospective study	640 patients	2.3% (15/640)	Brachiocephalic trunk (12/15); right CCA (2/15); aortic arch (1/15)	Anterior to trachea, to inferior thyroid pole	Preoperative CT angiography recommended before thyroid/parathyroid, tracheal, and mediastinal surgery	Not mentioned
Yohannan et al., 2019 [7]	India	Case report (cadaveric)	1 cadaver, male, ~60 yr, South Asian	Cited: 0.4–12.2%	Right subclavian artery close to vertebral artery origin — extremely unusual	Oblique superficial lateral-to-medial course; anterior to trachea (terminal branches only); to inferior right pole, isthmus, and both lobes. Bilateral ITA absence.	Bilateral ITA absence — high suspicion warranted for superficially running vessels in neck root. Risk in thyroidectomy, tracheostomy, and neck surgery.	TIA coursed considerably anterior to both RLNs — low RLN risk in this specific configuration
Yajima et al., 2021 [25]	Japan	Clinical case report (trauma)	1 patient, male, 91 yr	Cited: 0.4–12.2%	Brachiocephalic artery (confirmed angiographically)	Anterior to trachea; descending branch extends into mediastinum	TIA injury from blunt chest trauma → massive mediastinal haematoma → TAE successful. Emergency physicians must consider TIA in differential diagnosis of mediastinal haematoma.	Not mentioned
Novakov and Delchev, 2023 [26]	Bulgaria	Case report (2 cadaveric cases)	2 cadavers	Cited: 0.4–12.2%	Case 1: middle thymothyroid artery from right CCA (not a TIA — distinct vessel supplying thyroid and thymus). Case 2: brachiocephalic artery (replacing bilateral absent ITAs)	Case 2: spiral course; diameter 4 mm; length 21 mm; divides into right branch (→ thymic artery + right ITA) and left branch (→ left ITA + isthmus branch)	Awareness crucial for preoperative imaging and avoiding iatrogenic complications in thyroid surgery. Case 1 also illustrates risk in carotid endarterectomy and interventional procedures.	Not mentioned
Totlis et al., 2023 [27]	Greece	Case report (cadaveric)	1 cadaver, male, 90 yr	Cited: 3.8%	Brachiocephalic artery before bifurcation into RCCA and SCA; coexisted with brachiocephalico-carotid trunk	5 branches (3 anterior + 2 posterior) over trachea; supplied thyroid, inferior parathyroid glands, and infrahyoid muscles	Multiple arterial TIA branches over trachea = extreme bleeding risk during tracheotomy/cricothyroidotomy. Preoperative ultrasound recommended before any anterior neck procedure when TIA is suspected.	Not specifically mentioned
Bonnici et al., 2023 [34]	USA	Clinical case report	1 patient, female, 30 yr, Graves disease	Cited: ~3.8%	Identified during angiographic mapping of left subclavian territory (specific origin not stated in text)	Enlarged TIA; large perfusion distribution across isthmus and left upper and lower thyroid lobes	Successful TIA embolization (polyvinyl alcohol) for thyroid storm in neutropenic patient (methimazole-induced). 60% reduction in thyroid perfusion. Safe and effective alternative to thyroidectomy in selected patients.	Not mentioned
Bhardwaj et al., 2023 [21]	India	CT angiography retrospective study	210 patients, Garhwal region, Sub-Himalayan	0.48% (1/210)	Brachiocephalic trunk	Not detailed	TIA infrequent but may cause severe haemorrhage if unrecognized during neck/thyroid surgery	Not mentioned
Gaydarski et al., 2023 [33]	Bulgaria	Case report (cadaveric)	1 cadaver, male, 76 yr, Caucasian	Cited: 3.3%	Left internal thoracic artery (rare — 1.9% of TIA cases)	Transverse (1.9 cm) then ascending/medial (4.8 cm), anterior to trachea, to inferolateral left thyroid lobe. Lumen: 3.6 mm.	Risk in thyroidectomy, tracheotomy, laryngeal transplantation, SETA, parathyroid surgery. TIA supplied left inferior parathyroid gland — inadvertent ligation risks postoperative hypoparathyroidism.	Not mentioned
Bunea et al., 2024 [23]	Romania	Cadaveric + CTA mixed study	290 cases (82 cadavers + 208 CTA); Romanian Caucasian population	5.52% overall (16/290); left 4.14%, right 1.38% (right only in female subjects)	Aortic arch: 93.75% of TIA cases (15/16); brachiocephalic trunk: 6.25% (1/16, female subject)	Left-sided predominance (75% of TIA cases); straight vertical or oblique ascending course anterior to trachea; diameter 2.1–4.0 mm (left from AA). Coexists with left ITA in 50% of TIMA cases.	Highly variable artery — knowledge essential for surgical interventions involving thyroid gland. TIA diameter larger than coexisting ITA confirms compensatory role.	Not mentioned
Benedict et al., 2024 [28]	USA	Case report (cadaveric)	1 cadaver (anatomy lab dissection)	Cited: ~4%	Brachiocephalic trunk	Four-pronged variant: 3 branches to thyroid, 1 branch descending into thorax supplying anterior pericardium and surrounding adipose tissue	Unique mediastinal extension — risk of mediastinal bleeding. Preoperative imaging recommended before neck procedures to prevent catastrophic haemorrhage.	Not mentioned
Jha et al., 2024 [20]	Nepal	Cadaveric cross-sectional study	35 cadavers	5.71% (2/35)	Brachiocephalic trunk (both cases)	Ascending to inferior thyroid pole; bilateral ITA absence in both cases	Knowledge of TIA crucial to prevent serious complications before and after neck surgery	Not mentioned
Yaman et al., 2024 [31]	Turkey	CT angiography case report	1 patient, male, 73 yr (incidental finding)	Not reported (single case)	Aortic arch	Not detailed	Incidental CT finding — TIA presence has clinical importance prior to neck surgeries and minimally invasive interventions	Not mentioned
Xu et al., 2025 [29]	China	Case report (cadaveric)	1 cadaver, male, 64 yr, Asian (Mongoloid)	Not reported (single case)	Brachiocephalic trunk (compensating for absent left ITA)	U-shaped course; main trunk ~16 mm; 3 branches: (1) descending to anterior mediastinum, (2) to lower right thyroid lobe, (3) to isthmus and left lobe. Diameter 2.54 mm at origin.	Compensates for absent left ITA. Susceptible to uncontrollable haemorrhage. Prone to injury during tracheotomy, cricothyroidotomy, and superior mediastinum interventions.	Branch of TIMA ascended parallel to left RLN; minimum distance ~5.26 mm — differs from classic anterior/posterior/inter-arterial RLN–ITA relationships; warrants specific surgical attention
Wang et al., 2025 [30]	China	Surgical case report	1 patient, female, 28 yr, papillary thyroid carcinoma	Cited: 3–12%	Bilateral TIA from brachiocephalic trunk — first case ever reported	Right TIA: to lower right lobe. Left TIA: to isthmus/left lobe. Confirmed by postoperative 3D CT reconstruction.	First bilateral TIA case. Not identified preoperatively despite contrast-enhanced CT. Intraoperative recognition critical. Total thyroidectomy + bilateral neck dissection performed safely.	No RLN injury postoperatively
Bhardwaj et al., 2025 [21]	India	CT angiography prospective study	105 patients (20 with AA variations), Himalayan belt	10% of patients with AA variations (2/20) = 1.9% of total cohort	Brachiocephalic trunk (both cases)	Not detailed	Awareness of AA variations important for endovascular procedures and cardiothoracic surgeries	Not mentioned

The prevalence of the TIA at the study level was significantly different among the included articles, with 0.1% in a large cohort of 1,000 patients CTA [19] and 5.71% in a cross-sectional cohort of cadavers [20]. In the larger imaging-based series, Esen et al. found the TIA in 2.3% of 640 patients undergoing CTA of the carotid, brachiocephalic trunk, being the most frequent point of origin [8]. In a retrospective study of 210 patients undergoing CTA in the sub-Himalayan belt of north India, Bhardwaj et al. identified an occurrence of only one TIA due to the brachiocephalic trunk, and the prevalence was at 0.48% [21]. The same group reported TIA identification in 1.9% of the total study population in a separate prospective CTA study of 105 patients in a study about the Himalayan belt, and 10% of the patients with recorded aortic arch variations [22]. In a cadaveric-CTA study by Bunea et al. that examined 290 cases in a Caucasian population of Romania, the prevalence of TIA was 5.52 [23]. Another interesting observation was that the aortic arch was the most common site of origin, as seen in 93.75% of cases of TIA (15/16), and a brachiocephalic tract was only seen in one case. In 75% of the TIA cases, left-sided preponderance was revealed, which is opposite to the right-sided bias of older anatomy literature [23]. In the other reports, the prevalence data were not formally counted by the respective authors, who reported individual cadaveric or clinical findings.

The arterial origin of TIA showed a significant variance in all the studies included. The most common reported origin, as per both the case report and single-centred series literature, was the brachiocephalic trunk [14,20-22,24-30] by far. The aortic arch was found to be the predominant origin of the largest mixed cadaveric-imaging series [20] and the only aortic origin reported in two other CTA series [19,31]. In a case reported the right common carotid artery was reported to be the TIA origin in a case that also had an aberrant right subclavian artery (ARSA) and a right non-recurrent laryngeal nerve [32]. The less common sources were the right subclavian artery near the origin of the vertebral artery in an oblique superficial course [7], and the left internal thoracic artery in a Caucasian male cadaver in Gaydarski et al. [33]. The first bilateral TIA was reported by Wang et al. with the two vessels simultaneously emerging out of the brachiocephalic trunk in one patient who had total thyroidectomy due to papillary thyroid carcinoma [30]. An anatomical aspect of the case described by Totlis et al. is of particular interest since, in the given dissection, the TIA separated into five separate branches, three anterior and two posterior, passing over the trachea, and innervating not only the thyroid gland and inferior parathyroid glands but also the infrahyoid muscles, and a brachiocephalico-carotid trunk was also present [27].

Concerning the course of the anatomy, all the reported cases confirmed the typical anterior tracheal position of the TIA, rising up along its origin to the inferior pole of the thyroid gland or the isthmus. Nevertheless, there were notable exceptions to this trend. Yohannan et al. reported a uniquely off-axis and shallowly running course where the artery traversed the neck laterally between the common carotid artery medially and the internal jugular vein laterally, then crossed over superficially over the common carotid to the right thyroid pole, a demonstration specifically cited as a caveat to surgeons operating on the neck, as such a vessel would not be expected in that position [7]. An independent report of a cadaver in China reported a TIA taking the shape of a U-shape and proceeding onward to race over the inferior thyroid territory, with a main trunk of about 16 mm in length with three branches at its end, one of which was 10.5 cm, in the inferior thoracic part of the mediastinum [29]. The diameter of the TIA was officially measured in Bunea et al.'s series (2.1-4.0 mm on the left side of the aortic arch) [23], in Gaydarski et al.'s case (lumen 3.6 mm) [33], and in Xu et al.'s case (2.54 mm at origin) [29]. In most of the other reports, the measurements of the vascularity were not recorded systematically.

One of the clinical and statistically significant results of the various studies that were incorporated was the presence of the TIA in the presence of the unilateral or bilateral absence of the inferior thyroid arteries (ITAs). In Yohannan et al.'s cadaveric report from South Asia, bilateral ITA was missing, with TIA as the only arterial supply to both lobes of the thyroid gland and the isthmus [7]. A cadaveric study in Bulgaria similarly captured a case where a huge TIA arising in the brachiocephalic trunk substituted the bilaterally absent ITA, providing all the inferior thyroid vascular bed [23]. Another cadaveric dissection reported one-sided absence of the left ITA and the TIA off the brachiocephalic as the only vascular substitute [26]. This compensatory effect of the TIA in the absence of the ITA has direct clinical implications in thyroidectomy because, during an unexpected ligation of a TIA as the primary, or sole, inferior thyroid supply, there is the possibility of devascularization of the thyroid remnant or, if the TIA is also the supply of the parathyroid glands, of postoperative hypoparathyroidism. It is exactly this arrangement that was reported in a Bulgarian cadaveric case report, which showed that the TIA branch could extend to the left inferior parathyroid gland and the necessity of preoperative vascular mapping in the suspected cases [33].

The connection between the TIA and the recurrent laryngeal nerve (RLN) was touched upon by a few of the included studies. The TIA of the cadaver in Yohannan et al.'s case was identified to pass significantly anterior to both the RLNs, and thus, the risk of the RLN being injured by the TIA was determined to be low specifically in the tracheo-oesophageal groove when the nerve was in its natural location [7]. A more challenging spatial arrangement was revealed in a more recent Chinese cadaveric dissection, where one of the branches of the TIA was found to run parallel to the left RLN at a minimum distance of 5.26 mm, a relationship which deviates from the classic anterior, posterior or inter-arterial courses that are typically described between the RLN and the inferior thyroid artery and for which specific surgical care is required in thyroid dissection [29]. The spatial relationship between the TIA and the RLN was not given in any other study, which is a significant gap in the current literature.

The TIA has a surgical importance in thyroid and neck surgery, and this has been covered in the included studies in different levels of detail. Barbetakis et al. paid particular attention to the threat of catastrophic haemorrhage when performing a substernal thyroidectomy, and that the digital dissection of mediastinal thyroid extensions can tear a hitherto unknown TIA, which may require immediate sternotomy [14]. The haemodynamic intensity of these is increased by the fact that the TIA has its origins in the high-pressure arteries like the brachiocephalic trunk or aortic arch. In a clinical report from Greece, TIA injury at the time of percutaneous dilatational tracheostomy caused severe haemorrhage; this was converted to open tracheostomy, and the bleeding was eventually contained by ligation, and the patient survived [13]. However, the authors clearly indicated that urgent sternotomy would have been necessary had the ligation failed, showing the fine line between effective treatment and a disastrous result when the TIA remains undiagnosed. In a Japanese clinical case report, blunt thoracic trauma-induced TIA injury was followed by a huge mediastinal haematoma, which was successfully managed through transcatheter arterial embolization (TAE), defining TAE as a safe and efficacious minimally invasive haemostatic approach in the given clinical scenario [25]. Bonnici et al. reported an entirely different clinical presentation of a success story of embolization of a significantly enlarged TIA to manage thyroid storm in a neutropenic patient in whom total thyroidectomy was contraindicated because of neutropenia caused by methimazole, further increasing the clinical relevance of this vessel in general surgical anatomy [34].

A common theme across many of the included case reports was the need for greater preoperative awareness of the thyroidea ima artery and, in selected cases, for preoperative vascular imaging. A large series in Turkey found that CTA delineates the arterial supply to the thyroid gland in the preoperative period without the need for invasive techniques, and it must be taken into consideration in the cases of suspected anatomical variation [8]. As emphasized by Wang et al. in their case report, even contrast-enhanced CT could not detect the anomaly prior to surgery, and it could be detected only retrospectively with three-dimensional (3D) reconstruction of the vessels, which underlines the weakness of conventional preoperative imaging and the importance of careful intraoperative anatomical delineation [30]. A cadaveric case report by a Greek anatomical group described several TIA branches crossing over the anterior trachea and supported the use of ultrasound-guided tracheostomy as a safer alternative when an anomalous TIA is suspected over the trachea, since real-time sonographic guidance may decrease the risk of inadvertent vascular trauma [27].

Risk of Bias Assessment

Of the 20 included studies, 19 were formally appraised with the appropriate JBI Critical Appraisal Tool, and one was retained in the synthesis but not subjected to formal appraisal [14], for the reason explained in the Methods. All 19 formally appraised studies were classified as low risk of bias. The 12 case reports obtained scores between 6/8 and 8/8, with the most common gap being item Q2 on patient or donor history, which is rarely available in cadaveric reports.

The two case series, both based on cadaveric material, were also classified as low risk, with scores of 7/10 [17] and 9/10 [29]. The five CTA-based studies, including the mixed cadaveric and imaging series by Bunea et al. [23], were classified as low risk, with scores between 6/8 and 7/8; the points lost in this group concerned mostly the absence of a formal multivariable confounder analysis. A complete summary of the assessment is given in Table 2. Overall, the methodological quality of the included literature is acceptable, although it should be remembered that the evidence base is dominated by individual case observations, which inherently limit any inference at the population level.

**Table 2:** Methodological quality and risk of bias assessment of the 20 included studies, using the JBI Critical Appraisal Tools. JBI (Joanna Briggs Institute) scoring thresholds applied: low risk of bias ≥ 6/8 for case reports and cross-sectional studies, ≥ 7/10 for case series; moderate risk 4 to 5 out of 8 (or 5 to 6 out of 10); high risk below 4/8 (or below 5/10) [16-18].

Study (author, year)	Study Type	JBI Critical Appraisal Tool	Score	Risk of Bias
Yohannan et al., 2019 [7]	Cadaveric case report	Case Reports (8 items)	7/8	Low
Esen et al., 2018 [8]	CT angiography cross-sectional	Analytical Cross-Sectional (8 items)	7/8	Low
Kamparoudi et al., 2016 [13]	Clinical case report	Case Reports (8 items)	8/8	Low
Barbetakis et al., 2014 [14]	Editorial eComment	Not formally appraised*	N/A	N/A
Karacan et al., 2014 [19]	CT angiography cross-sectional	Analytical Cross-Sectional (8 items)	7/8	Low
Jha et al. 2024 [20]	Cadaveric case series	Case Series (10 items)	7/10	Low
Bhardwaj et al., 2023 [21]	CT angiography cross-sectional	Analytical Cross-Sectional (8 items)	6/8	Low
Bhardwaj et al., 2025 [22]	CT angiography cross-sectional	Analytical Cross-Sectional (8 items)	6/8	Low
Bunea et al. 2024 [23]	Mixed cadaveric and CTA series	Analytical Cross-Sectional (8 items)	6/8	Low
Lovasova et al., 2017 [24]	Cadaveric case report	Case Reports (8 items)	7/8	Low
Yajima et al., 2021 [25]	Clinical case report	Case Reports (8 items)	8/8	Low
Novakov and Delchev, 2023 [26]	Cadaveric case report	Case Reports (8 items)	7/8	Low
Totlis et al., 2023 [27]	Cadaveric case report	Case Reports (8 items)	7/8	Low
Benedict et al., 2024 [28]	Cadaveric case report	Case Reports (8 items)	6/8	Low
Xu et al., 2025 [29]	Cadaveric case report	Case Reports (8 items)	8/8	Low
Wang et al., 2025 [30]	Clinical case report	Case Reports (8 items)	8/8	Low
Yaman et al., 2024 [31]	CT angiography case report	Case Reports (8 items)	6/8	Low
Natsis et al., 2016 [32]	Cadaveric case series	Case Series (10 items)	9/10	Low
Gaydarski et al., 2023 [33]	Cadaveric case report	Case Reports (8 items)	7/8	Low
Bonnici et al., 2023 [34]	Clinical case report	Case Reports (8 items)	8/8	Low

Discussion

The current systematic review is a synthesis of evidence provided by 20 studies published between 2014 and 2025, consisting of cadaveric dissections, CTA, and case reports, and represents the up-to-date synthesis of the prevalence of TIA, anatomy of the thyroid gland, and clinical importance. The prevalence rates in the studies incorporated were as low as 0.1% in a large CTA cohort [19] and as high as 5.71% in a cadaveric series [20], which is not surprising given the known methodological difference between dissection-based and imaging-based detection. Such a gap was specifically quantified by Yurasakpong et al. [5], whose systematic review and meta-analysis not only provided an overall prevalence of adult TIA of about 3.3%, but also showed a high rate of cadaveric studies over CT-based investigations due to the higher resolution of direct dissection compared with routine angiographic methods in detecting small-calibre vessels. Another drawback of the imaging-based investigations is the ethnic and population level variations, since the reported higher prevalences of TIA in Asian cadaveric and surgical series [29,30] in comparison with the European CTA series [19,23] are consistent with the study of Toni et al. [4], who found statistically significant differences in the TIA prevalence among the Asian and Caucasian groups.

Among the most clinically relevant results of this review is the high and underestimated difference in the arterial source of the TIA. In classical anatomy, the most important source is the brachiocephalic trunk, and that is substantiated by most of the case reports in our series, as well as by the literature on the thyroid artery variation generally [35]. This assumption is, however, called into question by the mixed cadaveric-CTA study by Bunea et al. in a Romanian Caucasian population, in which the TIA originated in the aortic arch in most of the cases, and the brachiocephalic trunk was observed as the origin in only one specimen [23]. It remains unclear whether this difference represents a true population-level variation in the development of the vascular supply or an artefact of imaging methodology. The aortic arch origin produces a longer, more clearly visible vessel that may be identified on cross-sectional imaging than a shorter brachiocephalic trunk origin, and larger comparative studies are needed to settle this question. The point that is undisputed is that the source of the TIA cannot be presumed in any particular patient, and that the aortic arch needs to be methodically assessed together with the brachiocephalic trunk when preoperative vascular mapping is conducted. The heterogeneity in origin is further demonstrated by an Indian cadaver study which reports a right subclavian origin [7], a Bulgarian cadaver dissection which showed a left internal thoracic artery origin [33], and the first bilateral TIA case in which both vessels were reported to have arisen at the same time as the brachiocephalic trunk [30]. The latter report is especially educative, not so much due to its anatomical novelty, but because the bilateral arrangement had not been identified on standard contrast-enhanced CT but was only later identified through 3D vascular reconstruction, which highlights the shortcomings of conventional preoperative imaging [30]. The same anatomical heterogeneity was well-established with respect to the superior and inferior thyroid arteries [35,36], and the TIA must be perceived as a component of a larger picture of vascular variability in the thyroid area and not a curious single exception.

The coexistence of the TIA with unilateral or bilateral absence of the inferior thyroid arteries, documented across several included studies [7,26,29], carries directly actionable implications for intraoperative decision-making. The realization of the sudden loss of either or both ITAs when a surgeon is performing thyroid dissection must invariably result in the active search for a compensatory TIA in the inferior thyroid region. The converse condition is also critical, and in case TIA is detected during surgery, the surgeon should be in a position to determine whether it is the only blood vessel supplying the inferior parathyroid glands, as presented in the Gaydarski case, whereby the TIA branch went straight to the left inferior parathyroid gland [33]. In such a setup, unintended ligation or thermal damage of the TIA would effectively revascularize the gland, and the patient would be prone to postoperative hypoparathyroidism, one of the most frequent and clinically relevant complications of thyroid surgery, in which reported rates range widely with surgical technique, resection extent, and parathyroid gland localization [37-39]. The danger of parathyroid devascularization is increased by the fact that, in total thyroidectomy, the inferior poles are dissected bilaterally, and there is very little room to allow anatomical error, and that TIA serving as the main inferior parathyroid supply on one of the sides only may be the difference between normal and permanently impaired parathyroid functioning [37,39].

The spatial relationship between the TIA and the RLN is one of the least explored aspects of TIA surgical anatomy. It is a significant gap, as RLN identification and preservation are the most essential technical goals of thyroid surgery, and as any new vascular formation in the area of the usual location of the nerve should be characterized carefully. In the study by Yohannan et al., the TIA passed significantly anterior to both RLNs in a set of cadavers [7], which may indicate a fairly secure spatial proximity when the nerve takes its typical tracheoesophageal course. Nevertheless, a recent Chinese cadaveric case stipulates a more challenging anatomical situation, where an offshoot of the TIA rose alongside the left RLN at a distance not less than 5.26 mm [29], a distance which falls squarely within the zone of inadvertent injury during blunt dissection or operation of energy-based instruments. This parallel arrangement (which, according to the authors, is fundamentally different from the traditional anterior, posterior, or inter-arterial orientations outlined concerning the RLN-ITA correlation) may not be evident without careful and systematic dissection. The risk is also increased in those situations in which TIA is associated with non-recurrent laryngeal nerve, as reported by Natsis et al. [32], because non-recurrent RLN types have atypical lateral courses, which are more susceptible to intraoperative damage, no matter what the vascular anatomy might be like [40]. All these observations can be interpreted to mean that the TIA-RLN relationship is not predictable and is not always benign, and formal assessment of this relationship should be a priority in future cadaveric and intraoperative studies.

The hemorrhagic risk, which comes with unrecognized TIA, is the most acutely life-threatening aspect of this anatomical variant, and the provided literature is a strong collection of clinical examples. Since the TIA is always a great vessel of a high-pressure great vessel, either the brachiocephalic trunk or aortic arch, or a branch of these, cutaneous side effects on the vessel result in arterial bleeding, which is fast, pulsatile, and may not be easily controlled due to the limited space in which the operation takes place: the inferior neck or the superior mediastinum. Barbetakis et al. write on the particular risk in substernal thyroidectomy and advocate blind digital dissection of the mediastinal thyroid extension, a procedure that is routine practice in substernal goiter surgery, lacerating a TIA that has not been detected preoperatively or during the cervical stage of the surgery [14]. The literature on substernal goiter has continuously described the necessity of sternotomy as an infrequent yet possibly inevitable complication of the procedure, and an unidentified TIA is one of the anatomical aspects that can initiate this escalation [41,42]. The case by Kamparoudi et al. [13] is no exception to this risk. The hemorrhage was controlled by surgical ligation after conversion to an open tracheostomy, and a sternotomy was narrowly avoided. A separate Greek cadaveric dissection study found as many as five TIA branches crossing the anterior trachea in a single specimen [27], which serves as a vivid reminder that even simple operations on the anterior neck may face a vascular arrangement contrary to the normal anatomical expectation. In any of these cases, the linking pin across all these cases is a lack of preoperative awareness, and intraoperative finding of a TIA cannot be handled as normal, and unexpected arterial hemorrhage in the inferior neck should incorporate TIA origin among the possible causes.

Another clinically significant aspect of TIA is that it can be deliberately embolized therapeutically. Two studies reported the effective application of the TAE to control TIA-linked hemorrhage: Yajima et al. [25] in the context of blunt thoracic trauma with massive mediastinal hematoma and Bonnici et al. [34] in a patient presenting with thyroid storm and neutropenia as a result of methimazole use, in which thyroidectomy was contraindicated. The latter case is especially educative in showing that a significantly enlarged TIA with an expansive territory of thyroid perfusion can be the major vessel serving the thyroid tissue and can be selectively embolized to result in a clinically significant level of hyperthyroid control. These are compatible with the increased literature on the viability and usefulness of thyroid arterial embolization as a less invasive and safer alternative to surgery in a selected group of patients with hyperthyroidism, nodular goiter, or pre-surgical volume reduction [43,44]. To the interventional radiologist intending to perform thyroid embolization, mapping of TIA anatomy before the procedure is not only an anatomical checkpoint but also a clinical requirement because the inability to identify and embolize an enlarged TIA with a large proportion of thyroid blood flow can nullify the end result of the process, whereas the inability to identify a TIA that ends in one of the mediastinal structures, as reported in one American cadaveric study [28], may lead to an undesirable non-target embolization with potentially dire outcomes.

Collectively, these results of this review aid the adoption of a more systematic method of preoperative vascular assessment among patients having thyroid or anterior neck surgery. CTA continues to be the most detailed modality of definition of the origin and progression of the TIA [8], and 3D vascular reconstruction provides significant anatomical information that is not always identified with the standard axial acquisitions [30]. In patients where CTA is either not available or not suitable, preoperative ultrasound imaging of the inferior neck, that is, the search for an anomalous midline vessel in the tracheal space, is a viable and easily available alternative that has actually been suggested to be specifically used as a risk stratifier prior to tracheostomy in cases of suspected TIA [27]. Practically, preoperative vascular evaluation is advised to be compulsory in patients with known absence of ITA or hypoplasia on regular imaging, in those with known aortic arch malformations, and in all patients with the purpose of substernal goiter surgery, extension of the thyroid mediastinally, and in all patients with recurrent thyroid disease in a field that was previously operated.

The clinical situations in which preoperative angiography or CTA substantially changes surgical decision-making extend beyond the thyroid gland itself and underline the value of routine vascular mapping in any cervico-mediastinal procedure. In the surgery of substernal or retrosternal goiter, 3D CTA reconstruction allows the surgeon to identify an aberrant vessel such as the TIA in advance, define its origin from a high-pressure source, and choose between a cervical and a transthoracic approach without being caught off guard during the operation [41,42]. In thyroidectomies performed on patients with documented absence of the ITA, or with aortic arch anomalies, preoperative CTA can reveal a compensatory TIA that may be the dominant supply to the lower parathyroid glands, and the dissection can be planned accordingly to preserve parathyroid function. In tracheal resection or anterior mediastinal operations, defining the pretracheal vascular landscape in advance prevents inadvertent injury to a vessel that crosses anteriorly to the trachea. Finally, in interventional radiology, prior arterial mapping is a prerequisite before any selective transcatheter thyroid arterial embolization, since the failure to recognise and selectively cannulate an enlarged TIA can both reduce the clinical efficacy of the procedure and put the patient at risk [44,45]. Taken together, these examples illustrate that preoperative vascular imaging in this setting is not a refinement of comfort but a determinant of the actual operative strategy whenever an anomalous arterial supply may be present.

This review does not lack limitations. The literature included is made up of case reports and small series, which, by virtue of their nature, only report a selection of rare anatomic configurations and cannot be used to make reliable estimates of prevalence at a population level. All 19 studies that could be formally assessed were rated as low risk of bias on the relevant JBI tool, and one editorial was retained as supportive material without a formal score. Validated quality appraisal instruments have a limited discriminatory value in purely anatomical and descriptive literature [45], so the uniformly low risk of bias rating across the included studies should be read as a sign of reasonable reporting completeness rather than as a guarantee of unbiased prevalence estimates at the population level. Two articles could not be retrieved in full text and were therefore not included in the synthesis. One of them [46] may have contained additional anatomical or clinical information that we were unable to include in the present review. The heterogeneity of the methodology of detection among included studies (cadaveric dissection, CTA, and intraoperative identification) does not permit meaningful quantitative synthesis and implies that prevalence numbers are inaccurate based on actual biological prevalence, but instead, on the basis of a certain methodology. Lastly, the relationship between RLN and TIA is generally not well defined through the literature included in the review, with formal spatial data being output of only two studies, which is a gap that future anatomical and intraoperative studies must fill.

## Conclusions

TIA is a rare but important vascular anatomical variation that shows great variability in its origin of the artery, anatomical course, and pattern of branching, and cannot be reliably predicted based on standard anatomical assumptions only. The fact that it coexists with the absence of the ITA and may be contributing to the supply of the parathyroid gland, as well as being a direct derivation of high-pressure mediastinal vessels, all add up to a risk profile far beyond mere anatomical interest. The fact that the branches of the TIA are highly sensitive to the presence of the RLN in some of the configurations, but are rarely reported in the existing literature, further emphasizes the significance of careful and systematic dissection in the inferior thyroid territory.

Surgical practitioners who work in the anterior neck, thyroidectomy, substernal goiter, tracheostomy, or mediastinal exploration should be vigilant of this form of artery and assume an unanticipated arterial structure in the inferior thyroid or suprasternal space as a possible TIA, until proven otherwise. Three-dimensional vascular reconstruction using preoperative CTA should be considered in high-risk surgical cases, and the use of intraoperative ultrasound guidance is a viable solution in cases where TIA is suspected but preoperative imaging was performed. Future studies should focus on bigger prospective series with systematic recording of the correlation of TIA-RLN, standardized morphometric measures, and creation of a validated preoperative imaging guideline for at-risk patient populations.
